# *Notes from the Field:* Measles Outbreak at a United States Immigration and Customs Enforcement Facility ― Arizona, May–June 2016

**DOI:** 10.15585/mmwr.mm6620a5

**Published:** 2017-05-26

**Authors:** Heather Venkat, Ahmed M. Kassem, Chia-ping Su, Clancey Hill, Evan Timme, Graham Briggs, Kenneth Komatsu, Susan Robinson, Rebecca Sunenshine, Manisha Patel, Diana Elson, Paul Gastañaduy, Shane Brady, Measles Investigation Team, Measles Investigation Team, Jessica Rigler, Cara Christ, Eugene Livar, Lisa Villarroel, Rosa Lira, Corey Tarango, Teresa Jue, Sara Imholte Johnston, Don Herrington, Karen Lewis, Harmony Duport, Peter Kelly, Krista Anheluk, Irene Ruberto, Jennifer Pistole, Kristen Herrick, Jabette Franco, Samuel Packard, Christopher Reimus, Marcela Salinas, Tammy Sylvester, Ron Klein, Karen Rose, Karen Zabel, Jennifer Adair, Marcus Castle, Bob England, Edith Lederman, Geri Tagliaferri, Jennifer Freiman, Bessie Padilla, Herman Auhl, Paul Rota, Carole Hickman, Jessica Leung, Sun Bae Sowers, Sara Mercader, William Slanta, Kathryn Fitzpatrick, Jessica Escobar

**Affiliations:** ^1^Epidemic Intelligence Service, Division of Scientific Education and Professional Development, CDC; ^2^Arizona Department of Health Services; ^3^Maricopa County Department of Public Health; ^4^Idaho Department of Health and Welfare; ^5^Division of Surveillance, Hazard Evaluations, and Field Studies, National Institute for Occupational Safety and Health, CDC; ^6^Pinal County Public Health Services District; ^7^Office of Public Health Preparedness and Response, Career Epidemiology Field Officer Program, CDC; ^8^Division of Viral Diseases, National Center for Immunization and Respiratory Diseases, CDC; ^9^U.S. Immigration and Customs Enforcement.; Arizona Department of Health Services (ADHS; Pinal County Public Health Services District, Florence, Arizona; Maricopa County Department of Public Health, Phoenix, Arizona; U.S. Immigration and Customs Enforcement; Division of Viral Diseases, National Center for Immunization and; Respiratory Diseases, CDC; Arizona State Public Health Laboratory, ADHS.

On May 25, 2016, a detainee at a U.S. Immigration and Customs Enforcement (ICE) detention center in Arizona who had been hospitalized with fever and a generalized maculopapular rash was confirmed to have measles by real-time polymerase chain reaction (rPCR). A second case of measles in a staff member was confirmed by rPCR the next day. The privately operated, city-contracted facility housed 1,425 detainees, and employed 510 staff members, including 95 federal ICE staff and 415 contract staff of four distinct employers. Outbreak control measures consisted of administration of measles-mumps-rubella (MMR) vaccine to 1,424 detainees housed at the facility during May 29–31 and isolation of the detainee patient and any additional detainee patients identified during their remaining infectious period (until 4 days after rash onset). Recommendations were made by federal, state, and local public health partners to exclude staff members with measles-compatible symptoms as well as exposed staff members without presumptive evidence of immunity to measles.*

Epidemiologic investigations by local and state health departments and CDC identified 31 total cases of measles in 22 detainees and nine staff members, with rash onsets occurring May 6–June 26 (Figure). Initial reports of rash illness among a few detainees were attributed to varicella (chickenpox) based on clinical presentation; some detainees also reported that they did not initially seek medical attention when they became ill, likely leading to the delay in diagnosing the first few cases of measles. The median detainee patient age was 34 years (range = 19–52 years), and the median staff patient age was 41 years (range = 22–49 years). Seven of the nine ill staff members reported receipt of at least 1 dose of MMR vaccine in the past, but no vaccination records were available at the time the outbreak was recognized. Three of the nine ill staff members received 1 dose of MMR vaccine 7–13 days before becoming ill, suggesting that exposure might have occurred before sufficient immunity developed from vaccination, because the incubation period for measles ranges from 7–21 days.^†^ On June 17 and June 21, MMR staff member vaccination clinics were conducted on-site. Two additional clinics were conducted on July 15 and July 19. Staff members were encouraged to obtain their immunization records and to bring them to the facility to be recorded. Federal personnel policies and contractual agreements that do not require staff members to be vaccinated and the initial unavailability of staff member vaccination records might have contributed to low participation in the first two staff member vaccination clinics; only 120 MMR doses were administered, and 202 (40%) staff members were still considered to not have evidence of measles immunity.

**FIGURE:**
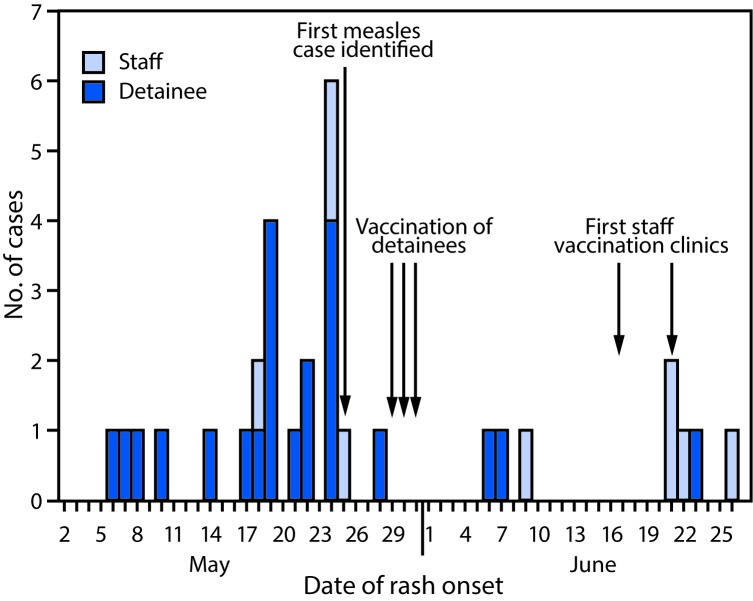
Confirmed measles cases (N = 31) in an immigration and customs enforcement facility, by date of rash onset and staff member/detainee status — Arizona, May 6–June 26, 2016

Reports of illness from personnel who had developed measles might have prompted other staff members to get vaccinated; by August 4, a total of 445 (87%) staff members were considered to have evidence of immunity, including 119 (23%) with documentation of receipt of 2 MMR doses before the start of the outbreak, 307 (60%) who had received 1 previous MMR dose and received a second dose during the outbreak, and 19 (4%) with serologic evidence of immunity. Although recommendations to exclude infectious staff members and nonimmune staff members suspected to have been exposed were made as soon as the outbreak was recognized, slow compliance with vaccination recommendations and incomplete implementation of exclusion recommendations, and restrictions on enforcing them, might have prolonged this outbreak.

Outbreak response is expensive and resource-intensive (*1*); specific strategies for measles prevention and control can be in place in advance to expedite and optimize containment in the event of an outbreak. First, persons working in congregate settings with populations that include people who have traveled internationally from measles-endemic regions or others whose immunity levels are unknown or difficult to assess should have documented evidence of measles immunity (*2*). Second, a means to quickly verify presumptive measles immunity among staff members in the event of occurrence of a case of measles can facilitate containment (*2*,*3*). Finally, contingency plans that allow for the exclusion of infectious staff members and exposed nonimmune staff members can prevent spread of measles (*3*,*4*). Adherence to these recommendations in high-risk settings, such as health care facilities, has been shown to limit transmission, optimize resources, and reduce costs (*4*).

Recommendations for implementing measles control policies for detention and correctional facilities, similar to those recommended in health care facilities, could be considered. If permissible, contractual and interagency agreements could include similar provisions, such as requiring MMR vaccination for staff members who work in detention facilities and do not have documented evidence of immunity.

## Footnotes

* Acceptable presumptive evidence of immunity against measles includes at least one of the following: 1) written documentation of adequate vaccination (≥1 dose of a measles-containing vaccine administered on or after the first birthday for preschool-age children and adults not at high risk; or 2 doses of measles-containing vaccine for school-age children and adults at high risk, including college students, health care personnel, and international travelers); 2) laboratory evidence of immunity; 3) laboratory confirmation of measles; or 4) birth before 1957.

† https://www.cdc.gov/vaccines/pubs/pinkbook/downloads/meas.pdf.
